# Evaluation of Machine Learning to Detect Influenza Using Wearable Sensor Data and Patient-Reported Symptoms: Cohort Study

**DOI:** 10.2196/47879

**Published:** 2024-10-04

**Authors:** Kamran Farooq, Melody Lim, Lawrence Dennison-Hall, Finn Janson, Aspen Hazel Olszewska, Muhammad Mamduh Ahmad Zabidi, Anna Haratym-Rojek, Karol Narowski, Barry Clinch, Marco Prunotto, Devika Chawla, Victoria Hunter, Vincent Ukachukwu

**Affiliations:** 1 Roche Data & Analytics Chapter (Data Science) Kaiseraugst Switzerland; 2 Genentech, Inc South San Francisco, CA United States; 3 Roche Products Ltd Welwyn Garden City United Kingdom; 4 Roche Global IT Solution Centre Warsaw Poland; 5 Roche Services & Solutions (Asia Pacific) Sdn Bhd Subang Jaya Malaysia; 6 Badger Software Sp z oo Wroclaw Poland; 7 Institute of Pharmaceutical Sciences of Western Switzerland University of Geneva Geneva Switzerland; 8 F Hoffmann-La Roche Ltd Basel Switzerland

**Keywords:** influenza, influenza-like illness, wearable sensor, person-generated health care data, machine learning

## Abstract

**Background:**

Machine learning offers quantitative pattern recognition analysis of wearable device data and has the potential to detect illness onset and monitor influenza-like illness (ILI) in patients who are infected.

**Objective:**

This study aims to evaluate the ability of machine-learning algorithms to distinguish between participants who are influenza positive and influenza negative in a cohort of symptomatic patients with ILI using wearable sensor (activity) data and self-reported symptom data during the latent and early symptomatic periods of ILI.

**Methods:**

This prospective observational cohort study used the extreme gradient boosting (XGBoost) classifier to determine whether a participant was influenza positive or negative based on 3 models using symptom-only data, activity-only data, and combined symptom and activity data. Data were collected from the Home Testing of Respiratory Illness (HTRI) study and FluStudy2020, both conducted between December 2019 and October 2020. The model was developed using the FluStudy2020 data and tested on the HTRI data. Analyses included participants in these studies with an at-home influenza diagnostic test result. Fitbit (Google LLC) devices were used to measure participants’ steps, heart rate, and sleep parameters. Participants detailed their ILI symptoms, health care–seeking behaviors, and quality of life. Model performance was assessed by area under the curve (AUC), balanced accuracy, recall (sensitivity), specificity, precision (positive predictive value), negative predictive value, and weighted harmonic mean of precision and recall (*F*_2_) score.

**Results:**

An influenza diagnostic test result was available for 953 and 925 participants in HTRI and FluStudy2020, respectively, of whom 848 (89%) and 840 (90.8%) had activity data. For the training and validation sets, the highest performing model was trained on the combined symptom and activity data (training AUC=0.77; validation AUC=0.74) versus symptom-only (training AUC=0.73; validation AUC=0.72) and activity-only (training AUC=0.68; validation AUC=0.65) data. For the FluStudy2020 test set, the performance of the model trained on combined symptom and activity data was closely aligned with that of the symptom-only model (combined symptom and activity test AUC=0.74; symptom-only test AUC=0.74). These results were validated using independent HTRI data (combined symptom and activity evaluation AUC=0.75; symptom-only evaluation AUC=0.74). The top features guiding influenza detection were cough; mean resting heart rate during main sleep; fever; total minutes in bed for the combined model; and fever, cough, and sore throat for the symptom-only model.

**Conclusions:**

Machine-learning algorithms had moderate accuracy in detecting influenza, suggesting that previous findings from research-grade sensors tested in highly controlled experimental settings may not easily translate to scalable commercial-grade sensors. In the future, more advanced wearable sensors may improve their performance in the early detection and discrimination of viral respiratory infections.

## Introduction

### Background

Between 2010 and 2020, an estimated 9 to 41 million annual illnesses were attributed to influenza infection in the United States [1]. Estimates of US annual hospitalizations ranged from 140,000 to 710,000 and deaths from 12,000 to 52,000 [1]. Early diagnosis and implementation of nonpharmaceutical interventions (eg, quarantine) are critical in preventing onward transmission and reducing the disease burden of influenza-like illnesses (ILIs). Recent data show that wearable devices (eg, fitness trackers and smartwatches) may help detect viral infection before symptoms develop and may provide an early warning system for viral illness [2-12].

In 2019 and 2020, approximately 29% of US adults were reported to use wearable devices [13], which range from fitness trackers that passively record heart rate (HR) and the number of daily steps to more sophisticated devices that can measure parameters such as sleep duration and quality, blood pressure, blood glucose, and oxygen saturation levels [14]. The wide availability and increasing popularity of wearable devices make them convenient, passive tools to record person-generated health data that could be harnessed to improve both individual and public health.

Wearable devices have the potential to detect the onset of illness and monitor disease progression or severity in patients infected with virus. In the future, this may allow people to be alerted to possible infection or the need to seek medical care in the early stages of disease [2-6]. Grzesiak et al [4] further showed that wearable devices may be able to predict the infection severity profile of a patient up to 24 hours before the onset of symptoms following exposure to the influenza virus or rhinovirus. The ability to predict illness severity may provide opportunities to discriminate between respiratory viral infections with more severe clinical presentation that carry a greater risk to public health, such as influenza and COVID-19, and those with a mostly mild clinical presentation, such as the common cold. Our previous analysis objectively characterized the “wearable phenotype” of individuals with ILI as well as those with confirmed influenza infection [15]. We demonstrated that before symptom onset, and throughout an ILI event, individuals experience reduced total daily steps, total active time, and sleep efficiency, as well as increased sleep duration and changes in resting HR (RHR) [15].

Here, we report the development and evaluation of a machine-learning model to detect laboratory-confirmed influenza infection based on wearable sensor and symptom data in the latent and early symptomatic periods (up to 1 day after symptom onset), using an extreme gradient boosting (XGBoost) classifier [16,17] in a cohort of symptomatic patients with ILI.

Machine learning offers a quantitative analysis of the data collected from wearable devices; XGBoost is an optimized distributed gradient boosting library that implements machine-learning algorithms, providing parallel-tree boosting [17]. XGBoost, a supervised machine-learning process, can be used to solve classification tasks, in which one can determine whether an instance is in a particular category by studying the features of that instance [17].

Using commercial wearable sensors (Fitbit [Google LLC]), it has previously been demonstrated that nationwide mobility (measured as total daily steps in a US population) decreased due to ILI symptoms and that ILI burden (determined by the difference in total daily steps) was associated with care-seeking behaviors, the number of workdays missed, and self-reported overall health [18]. Another study showed that abnormalities in RHR and sleep duration, measured by wearable sensors, could be leveraged to predict the real-time incidence of ILI [19]. Recently, wearable sensor data have also been used to assess physiological signs associated with COVID-19 [5,10,12,20-26].

### Objective

The objective of our analysis was to determine the ability of an XGBoost model to distinguish between participants who are influenza positive and influenza negative during the latent and early symptomatic periods of ILI (days –4 to +1). Wearable and symptoms data were used, gathered from 2 independent studies, FluStudy2020 and the HTRI study (NCT04245800); the former was used for training, testing, and validation, and the latter was used as a secondary holdout set for evaluation.

## Methods

### Study Design and Participants

#### Overview

This prospective observational cohort study evaluated the ability of machine-learning algorithms to distinguish between participants who are influenza positive and influenza negative in a cohort of patients with ILI. Analyses were conducted using wearable sensor (activity) data and self-reported symptom severity data from participants enrolled in FluStudy2020, with an influenza diagnostic test result from a self-administered kit [15,27]. Data from the HTRI study were used as an independent holdout set. All participants provided written consent.

The XGBoost model was used to classify whether a participant was influenza positive or negative based on 3 models using symptom-only data, activity-only data, and combined symptom and activity data. Other models were not assessed with these data based on previous internal analyses with different data, in which H2O AutoML was used to train and tune various models; XGBoost was found to be the best-performing model. Participant variables, including age, gender, BMI, and month in which the participant conducted an at-home influenza test, were considered before the Boruta feature selection algorithm was applied to the activity-only and combined symptom and activity models. The symptom-only model included all participant variables. The XGBoost model was assessed for its early detection of influenza infection in the FluStudy2020 training, validation, and test sets, as well as the HTRI secondary holdout set, using the following metrics: balanced accuracy, recall (sensitivity), specificity, precision (positive predictive value), negative predictive value, weighted harmonic mean of precision and recall (*F*_2_) score, and area under the receiver operating characteristic curve (AUC ROC). Calibration plots and feature importance plots were generated for each of the 3 models. A model evaluation schematic is shown in Figure S1 in Multimedia Appendix 1.

#### Data Collection and Preprocessing

The HTRI study and FluStudy2020 were conducted by Evidation Health in adults in the United States between December 2019 and October 2020. Participants in each study were aged ≥18 years, lived in the United States, and owned and were willing to wear a Fitbit device during the day and during sleep for the duration of the study. Full inclusion and exclusion criteria are shown in Table S1 in Multimedia Appendix 1. Steps, HR, and sleep data were collected through continuous passive monitoring via the participants’ Fitbit devices. Participants also completed daily surveys of whether they experienced influenza symptoms in the past 24 hours, self-reported ILI symptom severity, health care–seeking behaviors, and quality of life. Biweekly and monthly surveys were used to capture influenza-related complication events and vaccination history. Participants reporting certain ILI symptoms were instructed to perform a self-administered influenza diagnostic test. Samples were returned to the laboratory for the confirmation of influenza by a highly sensitive reverse transcription polymerase chain reaction test. The primary assessment of data from the 2 studies, including the removal of physiologically implausible data or null estimates, has been described previously [15]. Missing data were automatically handled by XGBoost by finding the best split direction when missing data were noted.

Participants with an influenza diagnostic test result were identified, and activity data were assessed for quality and completeness for each participant day. Step data were considered valid if the participant had at least 10 hours of step wear time [28,29] or if they had a valid HR day. HR data were considered valid if they included a minimum of 600 minutes (10 hours) of HR measurements and if a Fitbit-estimated RHR measure was available for that day. Sleep data were considered valid if nonzero and nonmissing total sleep minutes were available for the day. Finally, any day with <10 hours of wear time was considered invalid.

The maximum self-reported severity of 8 symptoms was analyzed: early fever, sore throat, cough, headache, muscle ache, chills, fatigue, and nasal congestion. In total, 41 activity features were analyzed, including RHR, total minutes asleep, the total number of steps, the proportion of the day that the participant spent being physically active (defined as ≥50 steps per minute), the maximum amount of activity the participant was able to complete within a single hour of the day, sleep efficiency score during main sleep, minutes in bed for the main sleep only of the day, the number of naps, total minutes in bed, the percentage of minutes with HR >1.5×RHR for the day, the proportion of minutes with nonzero steps out of the total minutes the device was worn, and mean RHR during main sleep. In addition, 29 HR variability (HRV) features were analyzed, derived from RHR captured during the participant’s sleep period.

#### Model Building and Optimization

Baseline predictors were assessed by including symptom-only features and activity-only features and then combining both features for the latent period to day 1 of ILI (days –4 to +1). This baseline model was built on the XGBoost classifier, which was selected as the machine-learning algorithm trained to detect influenza due to its scalability, regularization, and ability to detect complex nonlinear relationships. Metric calculations were based on a previously published study [5]:



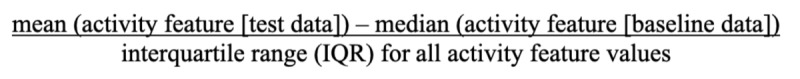



where test data represents ILI days –4 to +1, which encompass the latent period (days –4 to –1; ie, the incubation period for influenza), ILI onset (day 0), and part of the early symptomatic period (day +1), and baseline data represents the participants’ healthy baseline data from 2 weeks before the latent period (days –18 to –5). The model was optimized using the Bayesian hyperparameter optimization algorithm, a Bayesian inference, and a Gaussian process to find the maximum value of an unknown function with minimal iterations. AUC ROC was the metric subject to optimization; 100 optimization trials were run per model (symptom-only, activity-only, and combined symptom and activity data; each model included participant variables). The parameters optimized were maximum number of trees: (2, 50); learning rate: (0.0001, 0.2); maximum tree depth: (2, 10); subsample ratio of training instances before growing trees: (0.2, 1.0); column subsample ratio at each level: (0.1, 1.0); column subsample ratio at each tree: (0.1, 1.0); column subsample at each node: (0.1, 1.0); maximum delta step allowed by each leaf output: (0, 10); minimum sum of instance weight needed in a child (subtree: [0.0, 10.0]); L1 regularization: (0.00001, 1); and L2 regularization: (0.00001, 100). To mitigate the imbalanced ratio of participants who were influenza positive to influenza negative, class weights were calculated and applied to the model to give greater weight to the minority influenza-positive class.

Given these differences in study design, the current analysis excluded participants who tested positive for influenza in the HTRI study but did not meet any of the FluStudy2020 criteria for populations with ILI. There was also a small subset of HTRI participants whose symptoms would have met the influenza test kit criteria for FluStudy2020 but from whom a sample was not collected because they did not meet the HTRI influenza test kit criteria. To combine the HTRI data with the FluStudy2020 data, it was necessary to verify that the HTRI participants’ self-reported illness dates (which were provided in the same recovery survey in which a health care visit was reported) aligned with the analysis-derived ILI event dates (which were created during the analysis of the daily survey responses). This permitted verification that only health care visits during the same illness period as the ILI event period were included in the analysis. HTRI participants were categorized as having made or not made a health care visit only if their self-reported ILI event period overlapped with the analysis-derived ILI event period.

### Model Validation and Evaluation

Stratified k-fold shuffle cross-validation (k=50) was used to ensure model performance was reliable and robust. Overall, 50 models were trained on different training and validation sets before being evaluated on a single test set and the HTRI data. Of the FluStudy2020 data, 64% were used for k-stratified splits consisting of 50 training sets and 16% were used for validation. The remaining 20% of the data were set aside as the testing set. External validation was performed using the HTRI data. The Boruta feature selection algorithm was applied to reduce the dimensions of the activity features to minimize the impact of noise and reduce overfitting.

Model performance was assessed using the following metrics: balanced accuracy, recall (sensitivity), specificity, precision (positive predictive value), negative predictive value, *F*_2_-score, and AUC ROC. The model performance results consisted of the AUC ROC curves and mean performance across each k-fold along with 95% CIs for the training, validation, and test sets. The distribution of positive and negative predictions for the aggregated performance (based on symptom features, activity features, and these features combined) was described using confusion matrices. The values in each confusion matrix comprised the mean across each fold with their respective 95% CIs. Feature importance analyses were performed for each model, with the most important features summarized in feature importance plots.

### Software

Analyses were performed using Python (version 3.7; Python Software Foundation); *xgboost* (version 1.5.2) was used for modeling, and *bayesian-optimization* (version 1.2.0) was used for hyperparameter optimization. Feature importance was determined using XGBoost’s built-in feature importance. Data processing and visualization were performed with *pandas* (version 1.3.4), *NumPy* (version 1.21.4), and *Matplotlib* (version 3.5.1) [30-32]. *Kedro* was used to build robust and scalable data pipelines [33]. Feature selection was performed with *Boruta* (version 0.3) [34]. Statistical analysis was performed with *SciPy* (version 1.7.3) [35]. Metric computation, k-fold data splitting, and class weight calculations were performed using *scikit-learn* (version 0.24.2) [36]. The Python package *hrvanalysis* [37] was used to derive HRV features.

### Ethical Considerations

The HTRI study and FluStudy2020 were conducted by Evidation Health, Inc. Institutional review board approval was given by WCG Clinical for both the HTRI study (study number: 1271380; tracking number: 20192965) and FluStudy 2020 (study number: 1271500; tracking number: 20193003). Participants were recruited from the Evidation consumer platform, a free application that allows members to earn compensation for completing surveys, sharing health activity data, and reading health articles. Individuals were given the opportunity to enroll into the study once they provided informed consent to participate study activities and for use of their data. Participants earned reward points, redeemable for money, as compensation for completing study activities. Reward points worth up to US $10 were available on completion of enrollment, and a maximum of US $109 could be earned over the course of the study, if all study activities were completed. The data used for analysis were deidentified; each participant enrolled in the study was coded with a unique participant identification number.

## Results

### Participants

FluStudy2020 had 925 participants, of whom 840 (90.8%) had activity data that met the data density criteria. Of these 840 participants, 639 (76.1%) were influenza negative and 201 (23.9%) were influenza positive (Figure S1 in Multimedia Appendix 1). The HTRI study had 953 participants, and activity data meeting the data density criteria were available for 848 (89%) participants. Of these 848 participants, 657 (77.5%) were influenza negative and 191 (22.5%) were influenza positive. Baseline demographics of participants included in the model evaluation are presented in Table 1. Most participants were female (764/840, 90.9% and 660/848, 77.8%) with mean ages of 37.4 (SD 9.6) years and 37.6 (SD 9.1) years for FluStudy2020 and HTRI, respectively. Distributions of age, BMI, and gender were balanced between the group that was influenza negative and the group that was influenza positive. The maximum self-reported symptom severities and wearable sensor data during ILI days –4 to +1 are shown in Table 2 and Table S2 in Multimedia Appendix 1, respectively.

**Table 1:** Baseline demographics of participants included in the model evaluation.

Characteristics			FluStudy2020 participants							HTRI^a^ participants				
			Overall (n=840)		Influenza negative (n=639)		Influenza positive (n=201)		Overall (n=848)			Influenza negative (n=657)		Influenza positive (n=191)
Age (y), mean (SD)			37.42 (9.59)		37.10 (9.35)		38.45 (10.25)		37.55 (9.10)			37.47 (9.15)		37.83 (8.95)
BMI (kg/m^2^), mean (SD)			31.25 (8.16)		31.42 (8.16)		30.72 (8.18)		30.64 (7.51)			30.64 (7.69)		30.61 (6.89)
**Region, n (%)**														
	Midwest	291 (34.6)		206 (32.2)		85 (42.3)		299 (35.3)			216 (32.9)		83 (43.5)	
	Northeast	139 (16.6)		109 (17.1)		30 (14.9)		134 (15.8)			99 (15.1)		35 (18.3)	
	South	257 (30.6)		198 (31)		59 (29.4)		239 (28.2)			191 (29.1)		48 (25.1)	
	West	153 (18.2)		126 (19.7)		27 (13.4)		176 (20.8)			151 (23)		25 (13.1)	
**Gender, n (%)**														
	Female	764 (91)		586 (91.7)		178 (88.6)		660 (77.8)			519 (79)		141 (73.8)	
	Male	71 (8.5)		48 (7.5)		23 (11.4)		186 (21.9)			136 (20.7)		50 (26.2)	
	Nonbinary	5 (0.6)		5 (0.8)		0 (0)		2 (0.2)			2 (0.3)		0 (0)	
**Race, n (%)**														
	Alaska Native, American Indian, Native Hawaiian, or other Pacific Islander	2 (0.2)		2 (0.3)		0 (0)		1 (0.1)			1 (0.2)		0 (0)	
	Asian	16 (1.9)		13 (2)		3 (1.5)		27 (3.2)			19 (2.9)		8 (4.2)	
	Black or African American	31 (3.7)		25 (3.9)		6 (3)		21 (2.5)			14 (2.1)		7 (3.7)	
	White	753 (89.6)		568 (88.9)		185 (92)		763 (90)			593 (90.3)		170 (89)	
	Multiple races	32 (3.8)		27 (4.2)		5 (2.5)		28 (3.3)			23 (3.5)		5 (2.6)	
	Other	6 (0.7)		4 (0.6)		2 (1)		8 (0.9)			7 (1.1)		1 (0.5)	

^a^HTRI: Home Testing of Respiratory Illness.

**Table 2:** Frequency of maximum self-reported symptom severity during ILI^a^ days –4 to +1.

Characteristics			FluStudy2020 participants							HTRI^b^ participants				
			Overall (n=840)		Influenza negative (n=639)		Influenza positive (n=201)		Overall (n=848)			Influenza negative (n=657)		Influenza positive (n=191)
Age (y), mean (SD)			37.42 (9.59)		37.10 (9.35)		38.45 (10.25)		37.55 (9.10)			37.47 (9.15)		37.83 (8.95)
BMI (kg/m^2^), mean (SD)			31.25 (8.16)		31.42 (8.16)		30.72 (8.18)		30.64 (7.51)			30.64 (7.69)		30.61 (6.89)
**Region, n (%)**														
	Midwest	291 (34.6)		206 (32.2)		85 (42.3)		299 (35.3)			216 (32.9)		83 (43.5)	
	Northeast	139 (16.5)		109 (17.1)		30 (14.9)		134 (15.8)			99 (15.1)		35 (18.3)	
	South	257 (30.6)		198 (31)		59 (29.4)		239 (28.2)			191 (29.1)		48 (25.1)	
	West	153 (18.2)		126 (19.7)		27 (13.4)		176 (20.8)			151 (23)		25 (13.1)	
**Gender, n (%)**														
	Female	764 (91)		586 (91.7)		178 (88.6)		660 (77.8)			519 (79)		141 (73.8)	
	Male	71 (8.4)		48 (7.5)		23 (11.4)		186 (21.9)			136 (20.7)		50 (26.2)	
	Nonbinary	5 (0.6)		5 (0.8)		0 (0)		2 (0.2)			2 (0.3)		0 (0)	
**Race, n (%)**														
	Alaska Native, American Indian, Native Hawaiian, or other Pacific Islander	2 (0.2)		2 (0.3)		0 (0)		1 (0.1)			1 (0.2)		0 (0)	
	Asian	16 (1.9)		13 (2)		3 (1.5)		27 (3.2)			19 (2.9)		8 (4.2)	
	Black or African American	31 (3.7)		25 (3.9)		6 (3)		21 (2.5)			14 (2.1)		7 (3.7)	
	White	753 (89.6)		568 (88.9)		185 (92)		763 (90)			593 (90.3)		170 (89)	
	Multiple races	32 (3.8)		27 (4.2)		5 (2.5)		28 (3.3)			23 (3.5)		5 (2.6)	
	Other	6 (0.7)		4 (0.6)		2 (1)		8 (0.9)			7 (1.1)		1 (0.5)	

^a^ILI: influenza-like illness.

^b^HTRI: Home Testing of Respiratory Illness.

### Assessment of the XGBoost Model for Influenza Prediction During ILI Days –4 to +1

**Figure 1:**
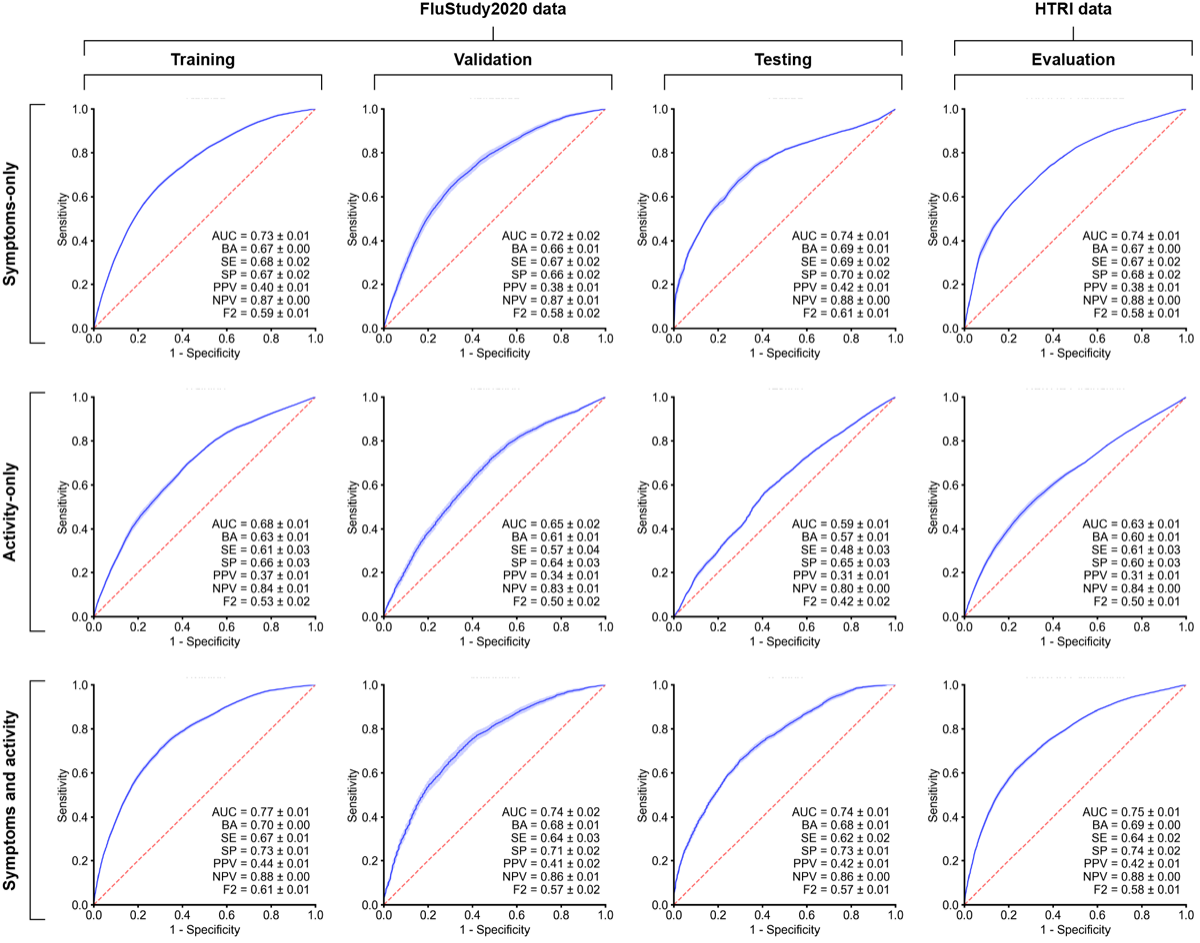
Receiver operating characteristic (ROC) curves for extreme gradient boosting (XGBoost) model discrimination between participants who were influenza positive and participants who were influenza negative for FluStudy2020 and Home Testing of Respiratory Illness (HTRI) data. XGBoost model performance was assessed for symptom-only data, activity-only data, and a combination of symptom and activity data. The mean performance across each k-fold and 95% CI for the training, validation, and test sets are presented. Mean values + or – the margin of error are shown for area under the curve (AUC), balanced accuracy (BA), sensitivity (SE), specificity (SP), positive predictive value (PPV), negative predictive value (NPV), and weighted harmonic mean of precision and recall (F2). The red line represents random guess, and the blue line represents mean ROC +95% and –95% CI.

XGBoost models informed by symptom-only data, activity-only data, or a combination of both symptoms and activity data were evaluated across training, validation, and test sets for FluStudy 2020. ROC curves and stratified k-fold cross-validation analyses for all models are presented in Figure 1, with confusion matrices shown in Figure S2 in Multimedia Appendix 1.

For the training and validation sets, the model trained on the combined symptom and activity data (training area under the curve [AUC]=0.77; validation AUC=0.74) consistently outperformed the models trained on the symptom-only data (training AUC=0.73; validation AUC=0.72) and activity-only data (training AUC=0.68; validation AUC=0.65; Figure 1). When applied to the FluStudy2020 test set, the model performance with the combined symptom and activity data was closely aligned with that of the symptom-only data (combined symptom and activity test AUC=0.74; symptom-only test AUC=0.74). We extended our evaluation to the HTRI study, where the model trained on combined symptom and activity data (evaluation AUC=0.75) outperformed the model trained on the symptom-only data (evaluation AUC=0.74), confirming the results of the FluStudy2020 training and validation sets.

**Figure 2:**
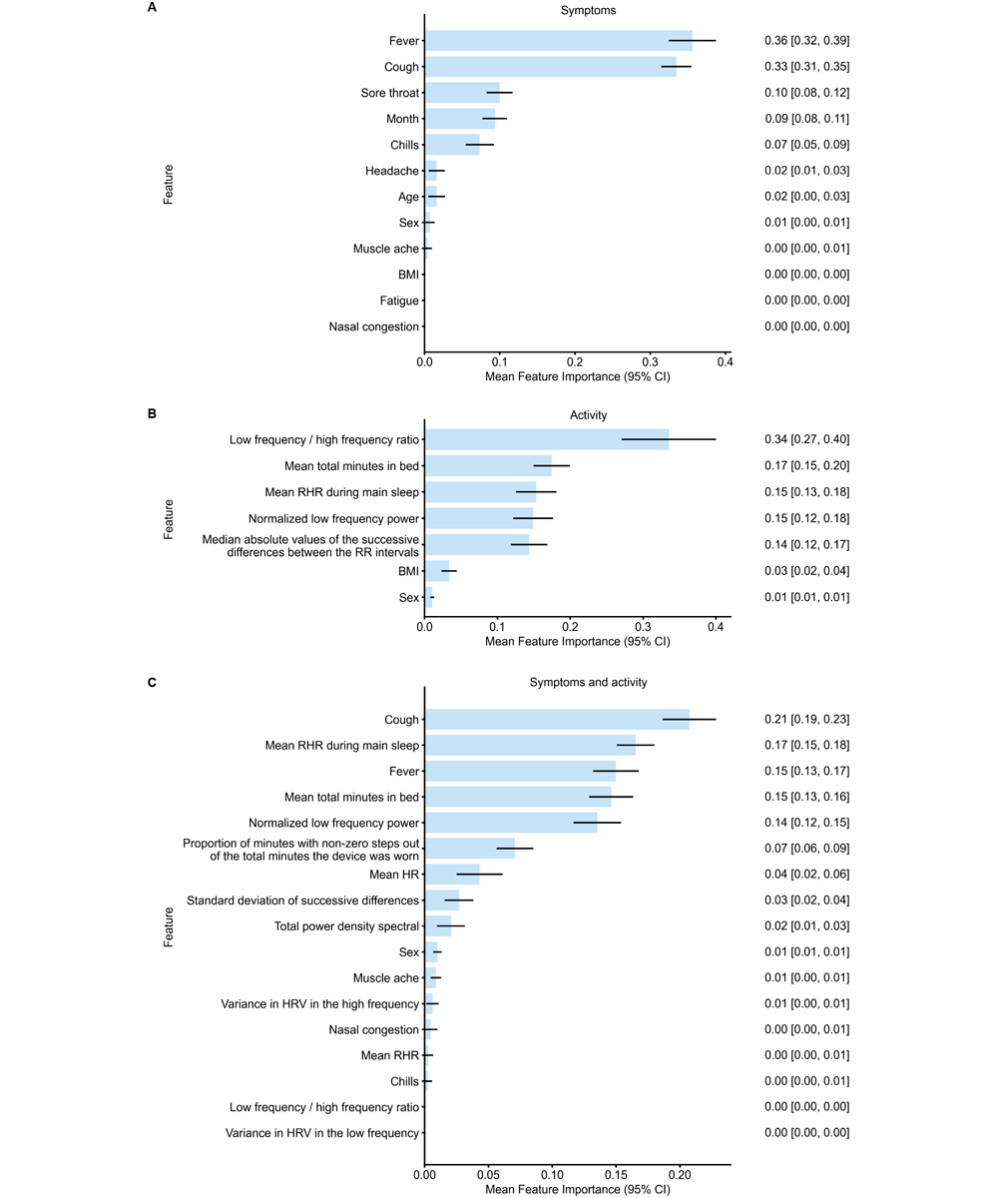
Feature importance plots for symptom-only data (A), activity-only data (B), and a combination of symptom and activity data (C). Values are presented as mean (95% CI). HR: heart rate; HRV: heart rate variability; RHR: resting heart rate; RR interval: the time elapsed between 2 successive R-waves of the QRS signal on the electrocardiogram.

**Figure 3:**
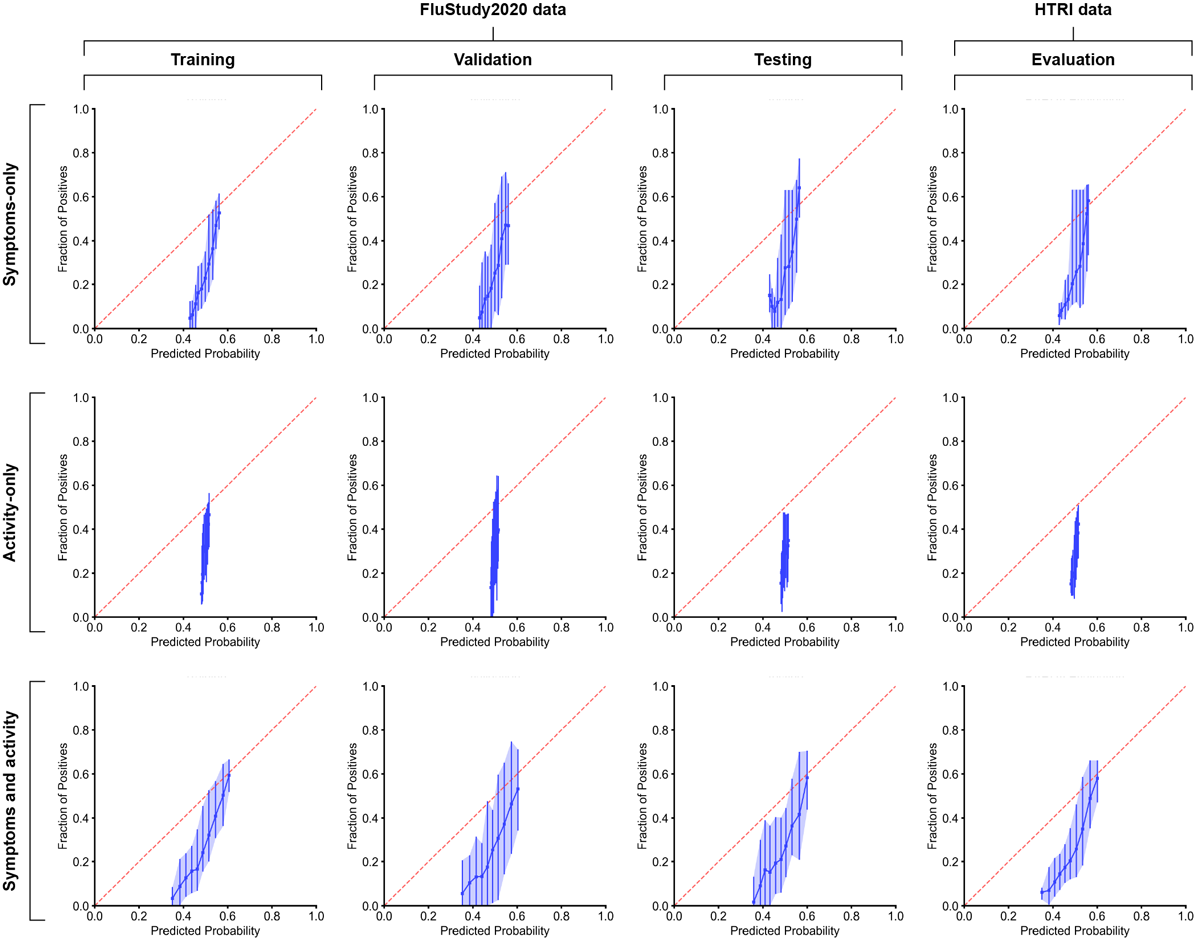
Calibration plots for FluStudy2020 and Home Testing of Respiratory Illness (HTRI) data, as assessed using symptom-only data, activity-only data, and a combination of symptom and activity data. The red dashed line represents perfect calibration, and the blue dots represent mean calibration +95% and –95% CI.

Feature importance plots for each model are presented in Figure 2. For the combined symptom and activity model, cough, mean RHR during main sleep, fever, and total minutes in bed were the most important, with mean feature importance values of 0.21, 0.17, 0.15, and 0.15, respectively. For the symptom-only model, fever, cough, and sore throat were the most important, with mean feature importance values of 0.36, 0.33, and 0.10, respectively. The heart low-frequency/high-frequency ratio, total minutes in bed, mean RHR during main sleep, and heart normalized low-frequency power were the top features influencing activity-only model predictions, with mean feature importance values of 0.34, 0.17, 0.15, and 0.15, respectively (Figure 2). Calibration plots highlighting the degree of correspondence between the estimated probability of influenza-positive cases and observed influenza cases for each model are presented in Figure 3.

## Discussion

### Principal Findings

To our knowledge, this is the largest study of commercial wearable sensors for the early detection of influenza incorporating the virological confirmation of influenza infection. The study was specifically designed to test, in the real world, the hypothesis generated in experimental settings that wearable sensor data may predict the onset of viral respiratory infection. For the combined symptom and activity model, the most important variables were cough, mean RHR during main sleep, fever, and total minutes in bed; for the symptom-only model, the most important variables were fever, cough, and sore throat; and for the activity-only model, the most important variables were heart low-frequency/high-frequency ratio, total minutes in bed, mean RHR during main sleep, and heart normalized low-frequency power. The best-performing machine-learning model for influenza detection was trained on the combined symptom and activity data and had a mean training AUC of 0.77. Model performance was validated on an independent data set (HTRI) not used for training, which yielded a mean AUC of 0.75. The accuracy of the combined machine-learning model was further confirmed by calibration plots for combined symptom and activity data, which were well calibrated compared with symptom-only or activity-only plots. Our model performance is significantly lower than that in other studies using machine-learning algorithms to predict influenza infection using wearable sensor data, which achieved accuracies of up to 94% [4,11]. However, these results were from small cohort (n=31 and n=20) challenge studies, where participants used research-grade wearable sensors and remained in controlled environments for up to 10 days following challenge with either influenza A virus subtype H1N1 or influenza A virus subtype H3N2 [4,11]. Grzesiak et al [4] noted that model accuracy was associated with both the knowledge of the timing and dosage of inoculation and the high-fidelity measurements of research-grade sensors. Our results suggest that findings from research-grade sensors tested in a highly controlled experimental setting may not easily translate to scalable low-fidelity commercial-grade sensors deployed in the real world.

With the FluStudy2020 training and validation sets, the best-performing model for influenza detection used a combination of symptom and activity features. However, in the FluStudy2020 test set, model performance was similar between the combined symptom and activity data model and the symptom-only data model. This implies the activity features do not significantly improve the model performance. In contrast, Quer et al [5], using similar methods to discriminate between symptomatic individuals who were positive or negative for COVID-19, found that a model combining symptom and sensor data performed significantly better than one considering symptoms alone (AUC 0.80, IQR 0.73-0.86 vs AUC 0.71, IQR 0.63-0.79). The different results observed may simply reflect differences between influenza and COVID-19, which have several nonoverlapping symptoms; notably, their model included data from onset day to day 7, whereas our model included data from day –4 to day 1. Our model restricted the data period, as the clinical utility of wearable sensors as an early warning tool for influenza would depend on their ability to detect infection early in its course, when the individual could take action to limit the spread or seek medical attention.

A key strength of our study is the laboratory confirmation of influenza in symptomatic patients using a highly accurate reverse transcription polymerase chain reaction test, which provides an accurate ascertainment of true positives and true negatives. Another strength of our study is that demographic, clinical, and Fitbit device data from a large, real-world population of >800 participants were used in model development. For the combined model (symptom and activity data), cough, total minutes in bed, and mean RHR during main sleep were the top 3 features influencing model predictions. Of the top 17 most important features influencing model predictions, 9 (53%) were HRV metrics. Deviations in HRV metrics have been associated with infection status and the severity of various bacterial and viral illnesses [4,7,10-12,22]. In addition, Hirten et al [22] showed that the mean of the SDs of normal-to-normal interval (the mean amplitude of the circadian pattern of the SD of the interbeat interval of normal sinus beats) was associated with a COVID-19 diagnosis, irrespective of symptomatology. Another study demonstrated that HRV acrophase and HRV midline estimating statistic of rhythm (MESOR) were among the most important predictors of COVID-19 infection, along with age and BMI [8]. Future analyses should consider the impact of biological and lifestyle factors, such as sex, menstrual cycle, and alcohol consumption, on HRV and other physiological features [9,10,38,39].

### Limitations

Limitations pertaining to the HTRI and FluStudy2020 study design have been discussed previously [15]. Notably, the very small numbers of African American participants, Asian participants, male participants, and participants aged ≥65 years in this cohort limit the generalizability of the model. The imbalances may be the result of differences in the likelihood of these populations to engage with digital health services; for example, women have been found to be more likely to use a mobile health app than men [40]. Participants were required to own a Fitbit device, which may predispose this cohort to exhibiting increased levels of activity and more health-conscious behaviors than the general population, which could limit the generalizability of the activity-based predictive models. A single device type (Fitbit) was used to minimize measurement errors that could arise from the use of multiple device types in study participants. However, this limits the generalizability of the findings, as several other device types are in widespread use. The self-reporting of symptoms in both studies is subjective and prone to recall bias. However, the results of influenza tests performed as part of the study were not provided to participants, which could have led to a differential recall of symptoms between participants who were influenza positive and influenza negative. Nevertheless, we cannot rule out participants’ awareness of their disease status through seeking routine care for their ILI outside of the study. In addition, symptom data were collected daily to minimize the risk of incomplete or inaccurate recall.

The studies included in this analysis were designed before the COVID-19 pandemic but were ongoing until October 2020. COVID-19 mitigation measures such as lockdown procedures may have impacted participants’ regular activities and influenza circulation during the period of these studies. Further implications of the COVID-19 pandemic have been discussed previously [15].

While our previous work demonstrates that the amplitude of wearable sensor deviations differs significantly between individuals who are influenza positive and those with ILI symptoms only, the symptom and activity features used in model development in this study are not unique to influenza infection [15]. Strict symptom criteria were used to define the symptomatic population with ILI, which may have led to the selection of a more severe symptomatic population and limited the ability to discriminate between participants who were influenza positive and participants who were influenza negative. Future studies with different study designs and less restrictive symptom eligibility criteria should investigate the ability of machine-learning algorithms to discriminate among a range of other common respiratory viral infections using symptomatic and wearable sensor data.

Finally, the validity and reliability of commercial wearable sensors in the measurement of steps, sleep, and HR have been a subject of debate. A systematic review including >150 publications found that Fitbit HR measurements were variable and tended toward underestimating HR [41]. The wearable devices used by participants in our study measured only steps, sleep, and HR. Future studies should investigate whether more advanced wearable sensors with more accurate accelerometers and including additional physiological measures, such as skin temperature and blood oxygen saturation, could improve the performance of commercial-grade sensors in the early detection and discrimination of respiratory viral infections.

### Conclusions

We demonstrate that a machine-learning algorithm combining symptomatic and commercial wearable sensor data during the latent and early symptomatic phases of ILI had moderate accuracy in detecting influenza in a large real-world cohort of symptomatic individuals with ILI, suggesting that previous findings from research-grade sensors tested in highly controlled experimental settings may not easily translate to scalable commercial-grade sensors deployed in the real world. The model maintained consistent performance across 2 distinct studies. The model was initially trained and evaluated on FluStudy2020 data and achieved comparable performance when validated on the HTRI data, affirming its generalizability. If machine-learning algorithms using commercial wearable sensors had strong predictive power and were validated, they may potentially play a role in public health surveillance and could prompt users to adopt infection-control behavior (eg, self-quarantine) and to seek early medical attention, if necessary. In the future, more advanced wearables measuring additional physiological parameters may improve the performance of wearable sensors in the early detection and discrimination of viral respiratory infections.

## Appendix

Multimedia Appendix 1 Supplementary tables and figures.

## Abbreviations

AUC ROC: area under the receiver operating characteristic curve
AUC: area under the curve
HR: heart rate
HRV: heart rate variability
HTRI: Home Testing of Respiratory Illness
ILI: influenza-like illness
MESOR: midline estimating statistic of rhythm
RHR: resting heart rate
XGBoost: extreme gradient boosting

## Data Availability

The Home Testing of Respiratory Illness (HTRI) data sets are available in the HTRI study research portal [42]. The data sets generated and analyzed during this study are available from the corresponding author on reasonable request.
