# Supplementary material for: Evaluation of Machine Learning to Detect Influenza Using Wearable Sensor Data and Patient-Reported Symptoms: Cohort Study
Source: J Med Internet Res. 2024 Oct 4;26:e47879. doi: 10.2196/47879 (PMC11489794; doi:10.2196/47879)

**Multimedia Appendix 1**

**Table S1.** FluStudy2020 and HTRI^a^ inclusion and exclusion criteria.

| **FluStudy2020** | | **HTRI** | |
| --- | --- | --- | --- |
| **Inclusion criteria** | **Exclusion criteria** | **Inclusion criteria** | **Exclusion criteria** |
| ≥18 years | Diagnosed with influenza within 3 months of the study start date | ≥18 years | Diagnosed with influenza within 3 months of the study start date |
| Lives in the US^b^ | Nasal surgery or procedure within 6 months of the study start date | Lives in the US | Currently enrolled in another influenza study being conducted by Evidation Health/Achievement studies |
| Speaks, reads, and understands English | Diagnosed with a bleeding disorder | Speaks, reads, and understands English |  |
| Owns and wears a FitBit |  | Owns a FitBit that collects heart rate data |  |
| Willing to wear a FitBit daily, during the day and during sleep |  | Willing to respond to a daily questionnaire for a 4-month period |  |
| Willing to respond to a daily questionnaire for a 4-month period |  | Has an iOS^c^ or Android smartphone/tablet that is capable of supporting the Audere flu@home app |  |
| Willing to participate in an at-home influenza testing procedure |  | Google Play Store compatible Android devices on Android 5.1 or later |  |
| Willing to submit pictures of medication labels for medication prescribed to treat or manage influenza |  | iOS devices on iOS 11 or later |  |
|  |  | Willing to download the Audere flu@home app |  |
|  |  | Willing to complete an at-home influenza testing procedure |  |

^a^HTRI: Home Testing of Respiratory Illness.

^b^US: United States

^c^iOS: iPhone Operating System.

**Table S2.** Wearable sensor (activity) feature metrics during ILI^a^ days –4 to +1.

| Wearable sensor (activity) metrics during ILI days –4 to +1 | FluStudy2020, mean (95% CI) | | HTRI^b^, mean (95% CI) | |
| --- | --- | --- | --- | --- |
|  | Influenza negative | Influenza positive | Influenza negative | Influenza positive |
| **HRV^c^ features** | | | | |
| Modified CSI^d^ | 14,111.93 (13,550.93-14,672.93) | 16,589.45 (15,311.72-17,867.19) | 10,814.64 (10,450.41-11,178.88) | 11,220.12 (10,537.81-11,902.43) |
| CSI | 34.46 (33.77-35.16) | 36.77 (35.57-37.98) | 31.44 (30.75-32.12) | 31.86 (30.69-33.04) |
| Cardiac vagal index | 3.57 (3.54-3.60) | 3.60 (3.55-3.64) | 3.52 (3.50-3.54) | 3.52 (3.48-3.56) |
| Variance in HRV in the high frequency | 4.06 (3.82-4.30) | 4.12 (3.70-4.54) | 3.75 (3.55-3.95) | 3.96 (3.51-4.40) |
| Normalized high-frequency power | 15.56 (15.52-15.60) | 15.61 (15.55-15.67) | 15.45 (15.41-15.48) | 15.48 (15.41-15.54) |
| Variance in HRV in the low frequency | 22.48 (21.07-23.89) | 22.75 (20.30-25.21) | 20.91 (19.75-22.06) | 22.14 (19.48-24.79) |
| Low-frequency/high-frequency ratio | 5.44 (5.42-5.45) | 5.41 (5.39-5.44) | 5.48 (5.47-5.50) | 5.47 (5.44-5.50) |
| Normalized low-frequency power | 84.44 (84.40-84.48) | 84.39 (84.33-84.45) | 84.55 (84.52-84.59) | 84.52 (84.46-84.59) |
| Total power density spectral | 138.63 (129.90-147.35) | 140.25 (125.06-155.43) | 128.92 (121.78-136.06) | 136.52 (120.10-152.94) |
| Variance in HRV in the very low frequency | 112.09 (105.01-119.16) | 113.37 (101.06-125.69) | 104.27 (98.48-110.05) | 110.43 (97.11-123.74) |
| HRV triangular index measurement | 8.66 (8.49-8.83) | 9.03 (8.75-9.30) | 8.15 (8.00-8.29) | 8.26 (8.00-8.53) |
| Percentage of minutes with HR^e^ >1.5×RHR^f^ for the day | 0.06 (0.06-0.07) | 0.07 (0.06-0.08) | 0.07 (0.06-0.07) | 0.08 (0.07-0.09) |
| Mean RHR during main sleep | 71.61 (70.79-72.42) | 72.87 (71.55-74.20) | 69.43 (68.72-70.13) | 70.45 (69.11-71.80) |
| SD of the projection of the Poincare plot on the line perpendicular to the line of identity | 2.96 (2.85-3.06) | 2.95 (2.78-3.12) | 2.90 (2.81-2.99) | 2.94 (2.76-3.12) |
| SD of the projection of the Poincare plot on the line of identity | 95.86 (93.10-98.63) | 102.11 (97.13-107.10) | 83.58 (81.62-85.53) | 84.49 (80.77-88.20) |
| Coefficient of variation equal to the ratio of SDNN^g^ divided by mean nni^h^ | 0.08 (0.08-0.08) | 0.09 (0.08-0.09) | 0.07 (0.07-0.07) | 0.07 (0.07-0.07) |
| Coefficient of variation of successive differences equal to the RMSSD^i^ divided by mean nni | 0.0047 (0.0046-0.0049) | 0.0048 (0.0046-0.0050) | 0.0045 (0.0044-0.0046) | 0.0046 (0.0044-0.0048) |
| Maximum HR | 95.25 (94.24-96.26) | 99.11 (97.52-100.69) | 85.30 (84.55-86.05) | 86.85 (85.41-88.29) |
| Mean HR | 72.19 (71.38-73.00) | 73.61 (72.30-74.93) | 69.77 (69.07-70.47) | 70.83 (69.48-72.17) |
| Mean of RR intervals^j^ (mean nni) | 857.35 (847.27-867.42) | 843.26 (827.44-859.07) | 882.15 (873.05-891.25) | 873.42 (856.42-890.41) |
| Median absolute values of the successive differences between the RR intervals | 863.51 (853.09-873.94) | 850.66 (834.18-867.13) | 884.26 (875.02-893.50) | 876.11 (858.94-893.28) |
| Minimum HR | 60.82 (60.08-61.55) | 61.08 (59.93-62.24) | 59.59 (58.95-60.22) | 60.40 (59.16-61.64) |
| Number of interval differences of successive RR intervals >20 milliseconds (nni 20) | 142.80 (137.48-148.12) | 151.23 (142.69-159.77) | 140.38 (135.67-145.10) | 138.85 (129.86-147.84) |
| Number of interval differences of successive RR intervals >50 milliseconds (nni 50) | 44.78 (42.34-47.22) | 48.09 (43.70-52.49) | 42.87 (40.65-45.08) | 43.37 (39.09-47.65) |
| Proportion derived by dividing nni 20 by the total number of RR intervals | 0.52 (0.50-0.54) | 0.51 (0.48-0.54) | 0.52 (0.50-0.54) | 0.52 (0.49-0.55) |
| Proportion derived by dividing nni 50 by the total number of RR intervals | 0.17 (0.16-0.18) | 0.17 (0.15-0.18) | 0.16 (0.15-0.17) | 0.17 (0.15-0.18) |
| Difference between the maximum and minimum nni | 361.51 (352.28-370.73) | 382.60 (366.46-398.74) | 313.82 (306.69-320.95) | 315.48 (302.11-328.85) |
| RMSSD | 4.18 (4.04-4.32) | 4.17 (3.93-4.41) | 4.10 (3.97-4.23) | 4.16 (3.90-4.42) |
| SDNN | 67.82 (65.87-69.78) | 72.24 (68.71-75.77) | 59.14 (57.75-60.52) | 59.78 (57.15-62.41) |
| SDSD^k^ | 4.18 (4.04-4.32) | 4.17 (3.93-4.41) | 4.10 (3.97-4.23) | 4.16 (3.90-4.42) |
| SD of HR | 6.13 (5.97-6.30) | 6.86 (6.53-7.20) | 4.74 (4.65-4.84) | 4.93 (4.75-5.12) |
| Mean RHR | 68.90 (68.16-69.64) | 68.84 (67.61-70.08) | 68.40 (67.74-69.05) | 67.98 (66.80-69.15) |
| **Sleep features** | | | | |
| Mean sleep duration | 432.32 (423.96-440.67) | 471.19 (458.19-484.19) | 429.51 (422.04-436.99) | 439.95 (424.71-455.18) |
| Mean sleep efficiency | 88.42 (87.36-89.47) | 90.76 (89.48-92.04) | 88.35 (87.34-89.37) | 88.02 (86.13-89.91) |
| Minutes in bed for the main sleep only of the day | 457.60 (450.33-464.87) | 484.73 (472.40-497.06) | 462.63 (456.69-468.57) | 461.90 (449.28-474.53) |
| Mean nap count | 0.27 (0.25-0.29) | 0.28 (0.24-0.32) | 0.21 (0.19-0.23) | 0.33 (0.29-0.37) |
| Mean total minutes in bed | 493.68 (486.26-501.11) | 526.61 (513.75-539.46) | 490.56 (484.49-496.64) | 506.42 (493.17-519.66) |
| **Steps and activity features** | | | | |
| Proportion of the day the participant spent being physically active (≥50 steps per minute) | 0.13 (0.13-0.14) | 0.13 (0.12-0.14) | 0.15 (0.14-0.15) | 0.15 (0.14-0.16) |
| Proportion of minutes with nonzero steps out of the total minutes the device was worn | 0.18 (0.17-0.18) | 0.17 (0.16-0.18) | 0.18 (0.18-0.19) | 0.18 (0.17-0.19) |
| Maximum amount of activity the participant was able to complete within a single hour of the day | 1812.75 (1719.70-1905.80) | 1753.13 (1595.91-1910.35) | 1930.59 (1825.67-2035.51) | 1915.84 (1738.62-2093.07) |
| Total number of steps | 7088.80 (6761.89-7415.71) | 7100.59 (6590.66-7610.52) | 7536.54  (7231.69-7841.39) | 7487.53 (6906.04-8069.03) |

^a^ILI: influenza-like illness.

^b^HTRI: Home Testing of Respiratory Illness.

^c^HRV: heart rate variability.

^d^CSI: cardiac sympathetic index.

^e^HR: heart rate.

^f^RHR: resting heart rate.

^g^SDNN: mean of the SDs of normal-to-normal interval.

^h^nni: normal-to-normal interval.

^i^RMSSD: square root mean of the sum of the squares of differences between adjacent normal-to-normal intervals.

^j^RR interval: the time elapsed between 2 successive R-waves of the QRS signal on the electrocardiogram.

^k^SDSD: SD of successive differences.

*Figure S1.* Model evaluation schematic. AUC: area under the curve; F2: weighted harmonic mean of precision and recall; HTRI: Home Testing of Respiratory Illness; ROC: receiver operating characteristics.


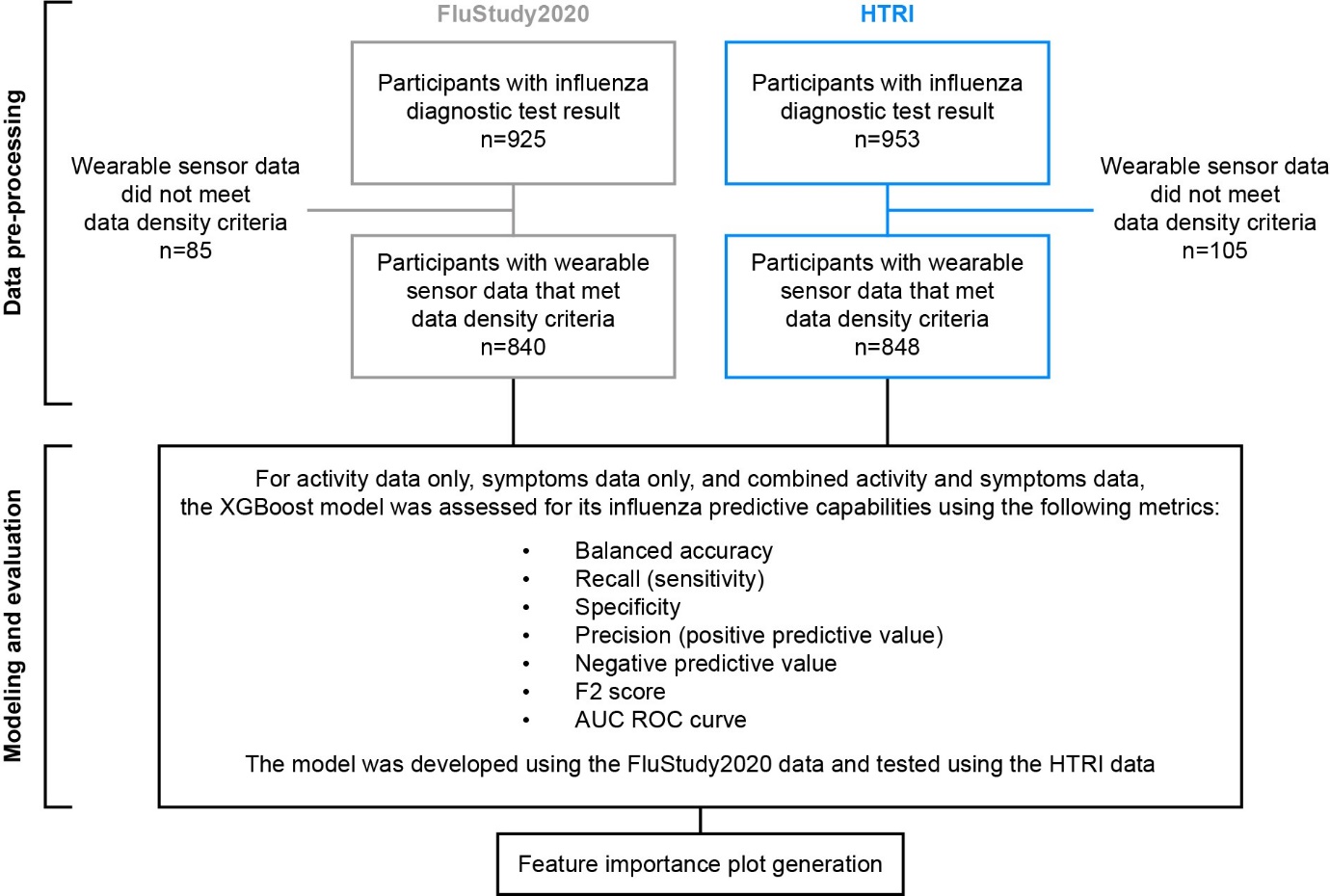


*Figure S2.* Confusion matrices for XGBoost model discrimination between influenza-positive and influenza-negative participants. XGBoost model performance was assessed for symptoms-only data, activity-only data, and a combination of symptoms and activity data. Mean values ± the margin of error are presented. HTRI: Home Testing of Respiratory Illness.


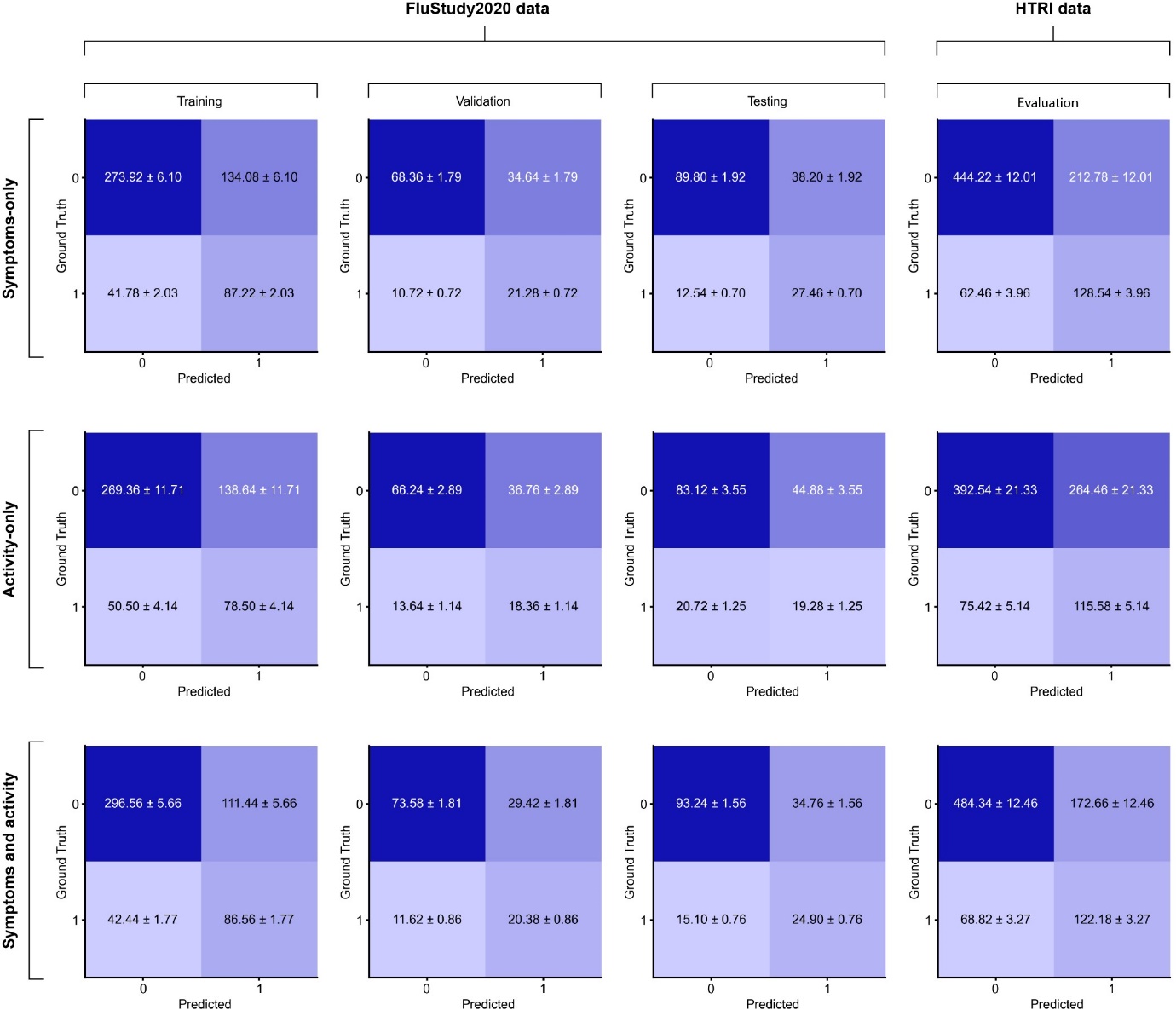

Supplement: Multimedia Appendix 1 [file jmir_v26i1e47879_app1.docx]
